# Ceftazidime–avibactam for multidrug and pandrug-resistant gram-negative infections in critically Ill children: a single-center pediatric ıntensive care experience

**DOI:** 10.1007/s00431-026-06862-1

**Published:** 2026-03-27

**Authors:** Merve Havan, Gül Arga, Yunus Emre Bülbül, Burak Özerdem, Eda Eyduran, Ayşen Durak Aslan, Nilay Penezoğlu, Hatice Belkıs İnceli, Duygu Öcal, Halil Özdemir, Ergin Çiftci, Tanıl Kendirli

**Affiliations:** 1 Division of Pediatric Intensive Care, Department of Pediatrics, Ankara University Faculty of Medicine, Ankara, Turkey; 2 Department of Pediatric Infectious Disease, Department of Pediatrics, Ankara University Faculty of Medicine, Ankara, Turkey; 3 Department of Pediatrics, Ankara University Faculty of Medicine, Ankara, Turkey; 4 Department of Microbiology, Ankara University Faculty of Medicine, Ankara, Turkey

**Keywords:** Ceftazidim-avibactam, Critically ill children, Carbapenem-resistant, *Enterobacteriaceae*, Antibiotic resistance

## Abstract

Carbapenem-resistant multidrug-resistant (MDR) and pandrug-resistant (PDR) Gram-negative infections represent a critical therapeutic challenge in pediatric intensive care units (PICUs), where effective treatment options are extremely limited. This study aimed to describe the real-world clinical outcomes and safety of ceftazidime–avibactam (CZA) in critically ill children with MDR/PDR infections. We conducted a retrospective observational study of pediatric patients aged 1 month to 18 years who received CZA for microbiologically confirmed MDR or PDR Gram-negative infections in a tertiary PICU between February 2021 and January 2025. Twenty-one critically ill children (median age 55 months, IQR 11–126) were included; two-thirds had underlying chronic conditions and 23.8% were immunosuppressed. *Klebsiella pneumoniae* was the predominant pathogen (85.7%). Microbiological clearance in follow-up cultures was observed in 85.7% of patients with available microbiological data, and clinical improvement was noted in most patients in this critically ill population. Infection-related mortality was 19% and was primarily associated with host-related factors, including younger age and immunosuppression. Resistance emergence during therapy was observed in one patient. Such resistance development during CZA treatment has been reported rarely in the literature and may involve mechanisms such as mutations in carbapenemase enzymes or alterations in bacterial permeability. In our study, resistance was detected during ongoing therapy in a single patient with *Klebsiella pneumoniae*, highlighting the importance of close microbiological monitoring in critically ill patients receiving prolonged treatment.

*Conclusion*: CZA was used as a salvage treatment in critically ill children with MDR and PDR Gram-negative infections, with favorable clinical and microbiological outcomes observed. These real-world data support its cautious incorporation into pediatric antimicrobial stewardship strategies in PICU settings.

**Table:** 

**What is Known:** • *Ceftazidime–avibactam is a promising agent against carbapenem-resistant Enterobacterales in adults.* • *Pediatric data remain limited, especially in critically ill children with MDR or PDR infections.*	
**What is New:** • *Ceftazidime–avibactam was used as salvage therapy for multidrug- and pandrug-resistant Gram-negative infections in critically ill children.* • *Clinical and microbiological response was achieved in a high-risk PICU population despite prolonged hospitalization and extensive prior antibiotic exposure.*

**What is Known:**

• *Ceftazidime–avibactam is a promising agent against carbapenem-resistant Enterobacterales in adults.*

• *Pediatric data remain limited, especially in critically ill children with MDR or PDR infections.*

**What is New:**

• *Ceftazidime–avibactam was used as salvage therapy for multidrug- and pandrug-resistant Gram-negative infections in critically ill children.*

• *Clinical and microbiological response was achieved in a high-risk PICU population despite prolonged hospitalization and extensive prior antibiotic exposure.*

## Introduction

The global emergence of multidrug-resistant (MDR) and pandrug-resistant (PDR) carbapenem-resistant bacteria has become a major threat in pediatric intensive care, where highly vulnerable patients often have few effective therapeutic options [1]. These organisms display resistance to most broad-spectrum antibiotics, including carbapenems, thereby severely narrowing treatment possibilities. Among carbapenemase-producing Enterobacteriaceae, *Klebsiella pneumoniae* carbapenemase (KPC) and OXA-48-type β-lactamases are particularly associated with severe, often life-threatening infections in children [2–4].

Ceftazidime–avibactam (CZA), a combination of the third-generation cephalosporin ceftazidime and the broad-spectrum β-lactamase inhibitor avibactam, has demonstrated promising activity against class A and D β-lactamase-producing organisms, including KPC and OXA-48 producers [5, 6]. While its clinical utility and safety have been well characterized in adult populations, data regarding its use in pediatric patients remain limited [5–8].

In Türkiye, CZA initially received temporary approval for pediatric use; however, subsequent regulatory changes have markedly restricted its availability for individuals under 18 years of age. Consequently, access to this antibiotic has become more limited in clinical practice. Given the growing prevalence of carbapenem-resistant pathogens and the scarcity of effective alternatives for children, real-world evidence on CZA use in pediatric intensive care settings is urgently needed. Recent systematic evidence has evaluated the clinical outcomes of CZA for infections caused by carbapenem-resistant Gram-negative bacteria. A systematic review and meta-analysis demonstrated improved clinical outcomes and reduced mortality compared with best available therapy in infections caused by OXA-48–producing Enterobacterales [9]. In addition, a recent systematic review focusing on pediatric populations reported generally favorable clinical and microbiological outcomes with acceptable safety profiles, although available pediatric data remain limited and are largely derived from small observational studies [10]. However, evidence from critically ill pediatric patients with pandrug-resistant infections, prolonged antimicrobial exposure, and complex device-associated infections remains scarce. In addition, access to newer antimicrobial agents such as CZA may be restricted in some healthcare systems, including Türkiye, making real-world data particularly valuable. Therefore, the present study aimed to describe the clinical outcomes and safety of CZA use in critically ill children treated in a tertiary PICU.

## Materials and methods

### Study design and setting

This retrospective descriptive case series study was conducted between February 2021 and January 2025 in a 20-bed tertiary PICU at a university hospital. The study included critically ill children aged 1 month to 18 years who were diagnosed with MDR or PDR carbapenem-resistant bacterial infections and received treatment with CZA.

Inclusion criteria were microbiologically confirmed infection due to MDR or PDR carbapenem-resistant *Klebsiella pneumoniae*, *Pseudomonas aeruginosa*, or *Acinetobacter baumannii*; the presence of clinical evidence of severe infection, such as sepsis, ventilator-associated pneumonia (VAP), or bloodstream infection; and CZA treatment for at least 48 h.

Exclusion criteria included initiation of CZA outside the PICU or before microbiological confirmation, treatment duration of less than 48 h, insufficient follow-up data, and terminal comorbidities expected to determine prognosis regardless of infection (e.g., end-stage organ failure or progressive malignancy). Neonates (age < 28 days) were not included in this study, as they are managed in a separate neonatal intensive care unit (NICU) at our institution with distinct patient characteristics and treatment protocols.

### CZA administration

CZA dosing followed pediatric age-specific recommendations based on available pharmacokinetic data. Infants under 3 months received 30 mg/kg of ceftazidime every 8 h intravenously; those aged 3 to < 6 months received 40 mg/kg every 8 h; and patients ≥ 6 months to < 18 years received 50 mg/kg every 8 h, up to a maximum of 2000 mg per dose. Avibactam was administered concurrently at the fixed formulation ratio. These regimens were based on available FDA pediatric pharmacokinetic and safety studies [11].

The decision to use CZA as monotherapy or combination therapy was individualized and determined by the treating physicians in consultation with pediatric infectious diseases and microbiology specialists. Combination therapy most frequently included meropenem or colistin, with tigecycline used in only two cases. Therapeutic drug monitoring for CZA was not routinely available in our institution. Therefore, dosing was performed according to age-based pediatric dosing recommendations and renal function. Source control procedures were implemented when clinically indicated as part of routine infection management. These included removal or replacement of infected central venous catheters and management of device-associated infections according to standard PICU practice.

### Microbiological methods and definitions

Hospital-acquired infections were confirmed through cultures obtained from blood, urine, wound, skin, stool, and tracheal aspirate samples. Blood cultures were processed using the Bactec FX system (Becton Dickinson, USA), and other samples were inoculated on standard media (Oxoid, UK). Antimicrobial susceptibility to CZA was assessed using the Phoenix 100™ system, Kirby–Bauer disk diffusion, and Etest MIC strips (bioMérieux, France), interpreted according to EUCAST criteria [12]. Carbapenem resistance was determined using routine phenotypic antimicrobial susceptibility testing performed in the clinical microbiology laboratory according to EUCAST criteria. Molecular confirmation of specific carbapenemase genes (e.g., KPC, OXA-48, NDM) was not performed. Carbapenem resistance was confirmed phenotypically using the carbapenem inactivation method (CIM). For *Acinetobacter baumannii* isolates, CZA susceptibility testing could not be analyzed due to the absence of established EUCAST or CLSI breakpoints; therefore, outcomes in these cases were evaluated based on clinical response rather than microbiological eradication. MDR and PDR classifications were defined according to the international consensus criteria proposed by Magiorakos et al. [13].

Diagnostic definitions followed international consensus guidelines. HAIs were defined in accordance with the CDC National Healthcare Safety Network (NHSN) surveillance definitions (January 2026 version), whereby infections were classified as healthcare-associated if the date of event occurred on or after the third calendar day of admission, with the day of admission counted as day 1 [14]. VAP was defined according to CDC/NHSN criteria [15], CRBSI according to CDC guidance [16], and sepsis and septic shock according to the Surviving Sepsis Campaign pediatric consensus [17].

### Data collection and outcome definitions

Demographic, clinical, and laboratory data were collected retrospectively from medical records. Variables included age, sex, comorbidities, type of infection, isolated microorganism, and antimicrobial susceptibility. Disease severity was assessed using PRISM III and PELOD scores. Outcome measures comprised PICU and hospital length of stay, microbiological clearance, mortality, and duration of clinical improvement under treatment. CZA therapy was initiated after microbiological confirmation of infection and susceptibility testing, based on the clinical judgment of the treating team in consultation with pediatric infectious diseases specialists. Infection-related mortality was defined as death occurring during active infection with microbiological and clinical evidence of ongoing sepsis or infection-related complications, as determined by multidisciplinary clinical review. Adverse events potentially attributable to CZA, including hepatotoxicity, nephrotoxicity, or hypersensitivity, were systematically monitored.

### Outcome definitions

Clinical response was defined as improvement or resolution of infection-related signs and symptoms during CZA therapy, including defervescence, hemodynamic stabilization, improvement in respiratory status, and decreased inflammatory markers when available, as documented in the medical record by the treating clinicians.

Microbiological eradication was defined as clearance of the causative pathogen in follow-up cultures obtained after initiation of CZA therapy. The timing of eradication was determined based on the first documented negative culture following a previously positive culture.

Infection-related mortality was defined as death occurring during the same hospitalization in the presence of ongoing infection or sepsis that was considered a major contributor to death based on clinical documentation, microbiological findings, and multidisciplinary review of the medical records.

###  Ethical approval

This study was conducted in accordance with the principles of the Declaration of Helsinki. Ethical approval was obtained from the Ethics Committee of the Ankara University Faculty of Medicine (Approval Number: 2025/681). Patient confidentiality was rigorously maintained throughout the study period.

### Statistical analysis

Statistical analyses were performed using IBM SPSS Statistics version 25.0 (IBM Corp., Armonk, NY, USA). Categorical variables were presented as frequencies and percentages. Continuous variables were summarized as mean ± standard deviation (SD) or median with interquartile range (IQR), depending on the data distribution (evaluated by visual inspection and the Shapiro–Wilk test). Comparisons between survivors and non-survivors were made using chi-square or Fisher’s exact tests for categorical variables, and Mann–Whitney U test for non-normally distributed continuous variables. A *p*-value < 0.05 was considered significant.

## Results

A total of 21 critically ill pediatric patients were included in the study. The median age was 55 months (IQR 11–126), and 10 patients (47.6%) were male. The median PRISM III and PELOD scores at admission were 19 (IQR 4–32) and 12 (IQR 2–19), respectively. Fourteen patients (66.7%) had underlying chronic conditions, including neuromuscular disorders (*n* = 6, 28.6%), post-transplant immunosuppression (*n* = 5, 23.8%), malignancy (*n* = 3, 14.3%), and other chronic conditions (*n* = 1, 4.8%). Before initiation of CZA, the median duration of hospitalization was 86 days (IQR 37–244), and the median PICU stay was 22 days (IQR 1–32) (Table 1).

**Table 1:** Baseline characteristics of all patients

Characteristics	Value
**Demographics**	
Age, median (IQR) 3 months–2 years ***n*** (%) > 2–6 years ***n*** (%) > 6–12 years ***n*** (%) > 12–18 years ***n*** (%) Male sex, *n* (%)	55 (11–126) 9 (42.8%) 2 (9.5%) 4 (19%) 6 (28.5%) 10 (47.6)
**Severity scores**	
PRISM III Score median (IQR) PELOD score median (IQR)	19 (4–32) 12 (2–19)
**Comorbidities and underlying conditions**	
Any underlying chronic disease, ***n*** (%) Chemotherapy use, ***n*** (%) Immunosuppressive drug use, ***n*** (%)	14 (66.7) 3 (14.3) 5 (23.8)
**PICU-related risk factors**	
Invasive mechanical ventilation, ***n*** (%) Duration of invasive MV, days, median (IQR) Central venous catheter, ***n*** (%) Duration of catheter use, days, median (IQR) VP shunt history, ***n*** (%)	19 (90.5) 30 (10–360) 21 (100) 67 (12–148) 5 (23.8)
**Prior antimicrobial exposure**	
Broad-spectrum antibiotic use, ***n*** (%) Duration before CZA, days, median (IQR) PICU stay before Ceftazidime-Avibactam median (IQR) Hospital stay before Ceftazidime-Avibactam median (IQR)	21 (100%) 76 (16–135) 22 (1–32) 86 (37–244)
**Antibiotic groups used before CZA, n (%)**	
Carbapenem Polymyxin Glycopeptide Aminoglycoside Fluoroquinolone Antifungal agents	21 (100) 21 (100) 20 (95.2) 19 (90.5) 11 (52.4) 18 (85.7)

*CZA* ceftazidim-avibactam, *MV* mechanical ventilation, *PICU* pediatric intensive care unit, *VP* ventriculoperitoneal

All patients (*n* = 21, 100%) had undergone invasive procedures and had prior antimicrobial exposure. Nineteen children (90.5%) required mechanical ventilation, with a median duration of 30 days (IQR 10–360). All patients had central venous catheters in place (median duration 67 days, IQR 12–148), and five patients (23.8%) had ventriculoperitoneal shunts. Previous antimicrobial therapies included carbapenems and polymyxins in 21 patients (100%), glycopeptides in 20 patients (95.2%), aminoglycosides in 19 patients (90.5%), fluoroquinolones in 11 patients (52.4%), and antifungals in 18 patients (85.7%). Chemotherapy and immunosuppressive therapy were administered in 3 patients (14.3%) and 5 patients (23.8%), respectively (Table 1).

Of the 21 infections, 16 (76.1%) were caused by PDR organisms and five (23.9%) by MDR strains. All infections were hospital-acquired and predominantly device-associated. *Klebsiella pneumoniae* was the most frequently isolated pathogen (*n* = 18, 85.7%), followed by *Acinetobacter baumannii* (*n* = 2, 9.5%) and *Pseudomonas aeruginosa* (*n* = 1, 4.8%). Because ceftazidime–avibactam has no established susceptibility breakpoints for A. baumannii, these cases were analyzed descriptively and interpreted as exploratory observations rather than evidence of antimicrobial efficacy. The most common infection types were CRBSI (*n* = 14, 66.7%) and VAP (*n* = 11, 52.4%). Other infection types included surgical site infections (*n* = 2, 9.5%), central nervous system infections (*n* = 2, 9.5%), and non-CRBSI bloodstream infections (*n* = 3, 14.3%). Nine patients (42.9%) required isolation due to colonization or infection with phenotypically identified carbapenem-resistant organisms. CZA therapy exceeded 14 days in 15 patients (71.4%). Mortality appeared similar between patients receiving ≤ 14 days and those receiving > 14 days of therapy.

A descriptive comparison was also performed between patients receiving CZA monotherapy (*n* = 7) and those receiving combination therapy (*n* = 14). Combination regimens most frequently included meropenem or colistin, with tigecycline used in two cases. Among patients with bloodstream infection, the duration of bacteremia was defined as the number of days from the first positive blood culture to the first documented negative culture. The median duration of bacteremia was 3 days (IQR 2–5) in the monotherapy group and 4 days (IQR 2–6) in the combination therapy group. Microbiological clearance in follow-up cultures was documented in 6 of 7 patients (85.7%) receiving monotherapy and 12 of 14 patients (85.7%) receiving combination therapy. Emergence of resistance during therapy was rare, occurring in one patient in the combination therapy group, whereas no resistance emerged among patients receiving monotherapy. Patients treated with combination therapy generally had more complex clinical courses, including prolonged mechanical ventilation and extensive prior antimicrobial exposure. Given the observational design, the small sample size, and potential confounding by indication, these findings are presented descriptively and should not be interpreted as evidence of superiority of one regimen over another.

A comparison between survivors (*n* = 11, 52.4%) and non-survivors (*n* = 10, 47.6%) is presented in Table 2. PRISM III and PELOD scores, duration of mechanical ventilation, and the frequency of septic shock were broadly similar between survivors and non-survivors. CRBSI was more frequently identified among survivors (*n* = 11, 100%) compared with non-survivors (*n* = 3, 30.0%). Non-survivors were younger (median age 6.2 months vs 72 months), and immunosuppression was more frequent among non-survivors (*n* = 3, 30.0%) compared with survivors (*n* = 2, 18.2%); however, neither comparison reached statistical significance.

**Table 2:** Infection and admission characteristics between survivors and non-survivors

Variable	Survivors (*n* = 11)	Non-survivors (*n* = 10)
Age (months), median (IQR)	64 (36–132)	60 (24–108)
Gender (Male), *n* (%)	6 (54.5%)	5 (45.5%)
PRISM III score, median (IQR)	18 (12–24)	21 (16–32)
Hospital stay (days), median (IQR)	86 (48–132)	72 (45–108)
PICU stay before CZA (days), median (IQR)	79 (56–108)	76 (54–96)
Underlying disease, *n* (%)	11 (100%)	10 (100%)
Immunosuppression (*n*)	2 (18.2%)	3 (30%)
Ventilator-associated pneumonia, *n* (%)	7 (63.6%)	4 (40%)
Catheter-related bloodstream infection, *n* (%)	11 (100%)	3 (30%)
Wound infection (*n*)	4 (36.4%)	1 (10%)
Meningitis, *n* (%)	0 (0%)	1 (10%)
Sepsis, *n* (%)	6 (54.5%)	5 (45.5%)
Septic shock *n* %	6 (54.5%)	7 (70%)
Bacteremia *n* (%)	11 (100%)	10 (100%)
*K. pneumoniae* isolated, *n* (%)	9 (81.8%)	9 (90%)
Acinetobacter isolated, *n* %	1 (9.1%)	1 (10%)
Pseudomonas isolated, *n* (%)	1 (9.1%)	0 (0%)
Isolation room used, *n* (%)	4 (36.4%)	5 (50%)
Colonized patient nearby, *n* (%)	11 (100%)	10 (100%)
CZA ≤ 14 days, *n* (%)	2 (18.2%)	4 (40%)
CZA > 14 days, *n* (%)	9 (81.8%)	6 (60%)
Post-treatment microbial growth, *n* (%)	1 (9.1%)	2 (20%)
Adverse effects (*n*)	0 (0%)	1 (10%)

*CZA* ceftazime-avibactam, *IQR* interquartile range, *PICU* pediatric intensive care unit

Clinical response was evaluated according to the predefined outcome criteria described in the Methods section and was observed in most patients during CZA therapy. Among patients with available follow-up cultures, microbiological eradication was documented in 12 of 14 cases (85.7%). Persistent growth was observed in three patients, primarily in respiratory specimens. In patients with *Acinetobacter baumannii* infections (*n* = 2, 9.5%), CZA was administered as part of a salvage treatment approach; a clinical response was observed in one patient (50.0%). Patients with infection-related mortality tended to have longer durations of mechanical ventilation and central venous catheterization and more frequent sepsis and septic shock.

A separate descriptive analysis focusing on infection-related mortality is presented in Table 3. Among the four infection-related non-survivors (*n* = 4, 19.0%), the median age was 6.2 months (IQR 2–11). All had microbiologically confirmed infections, including VAP, meningitis, or CRBSI. The causative organisms were *Klebsiella pneumoniae* (*n* = 3, 75.0%) and *Acinetobacter baumannii* (*n* = 1, 25.0%). The median duration of CZA therapy was 18 days (IQR 7–32). Median PICU stay was 57 days (IQR 21–145), and total hospitalization was 74 days (IQR 28–165). Three patients (*n* = 3, 75.0%) were immunosuppressed.

**Table 3:** Clinical and laboratory characteristics of patients with and without ınfection-related mortality

Parameter	Survivors (*n* = 17)	Non-survivors (*n* = 4)
Age (months), median (IQR)	36 (18–84)	42 (24–96)
Gender (Male), *n* (%)	9 (52.9%)	2 (50.0%)
PRISM III score, median (IQR)	26 (19–29)	25 (22–30)
PELOD score, median (IQR)	12 (8–19)	14 (10–21)
MV duration (days), median (IQR)	21 (14–33)	30 (22–36)
PICU stay (days), median (IQR)	64 (39–89)	79 (67–93)
CRP (mg/L), median (IQR)	90 (76–125)	120 (100–145)
PARDS presence, *n* (%)	6 (35.3%)	2 (50.0%)

*MV* mechanical ventilation, *PICU* pediatric intensive care unit, *CRP* C-reactive protein, *PARDS* pediatric respiratory distress syndrome

In one patient with *Klebsiella pneumoniae* infection, resistance to CZA was detected during treatment. The resistant isolate was identified in the fifth follow-up blood culture obtained on day 18 of CZA therapy. Carbapenem resistance was confirmed phenotypically using the CIM in the clinical microbiology laboratory. Baseline and follow-up MIC values for CZA were not available in the microbiology reports, and molecular characterization of resistance mechanisms was not performed. Following detection of resistance, combination antimicrobial therapy covering Gram-negative and Gram-positive pathogens was initiated; however, the patient died due to progressive multiorgan dysfunction secondary to severe infection.

Despite targeted antimicrobial treatment, all four patients died from progressive multiorgan dysfunction secondary to severe infection. Among these four patients, microbiological eradication was achieved in three, with documented clearance of the causative pathogen on follow-up cultures. In one patient, persistent positive cultures were observed, and resistance to CZA emerged during therapy. One adverse event was documented during CZA therapy. A single patient developed mild hand tremor during treatment. The event was transient, did not require discontinuation of CZA, and resolved without specific intervention. No severe drug-related adverse effects were observed.

## Discussion

In this study, we present a single-center pediatric intensive care experience with CZA, highlighting its role in the management of MDR and PDR gram-negative infections, most commonly caused by *Klebsiella pneumoniae*. Overall clinical and microbiological outcomes appeared favorable, with substantial microbiological clearance and clinically meaningful recovery observed in most patients, consistent with recent pediatric reports despite limited therapeutic alternatives [5–7].

Recent systematic reviews have reported generally favorable clinical outcomes and acceptable safety profiles for CZA in resistant Gram-negative infections, with pooled analyses suggesting potential mortality benefits compared with alternative therapies [9, 10]. However, most available evidence derives from adult cohorts, and pediatric data remain limited. Our study therefore contributes additional real-world observations from a tertiary PICU population, including patients with a high proportion of pandrug-resistant infections and prolonged antimicrobial exposure.

The predominance of *K. pneumoniae* and device-associated infections reflects the invasive nature of the PICU and the high burden of hospital-acquired infections in this population. Bloodstream infections were the most frequent infection type, and early recognition with timely source control may have contributed to more favorable outcomes, whereas pneumonia and central nervous system infections were associated with a more complicated clinical course [18].

Evidence supporting ceftazidime–avibactam activity against *Acinetobacter baumannii* remains very limited. Evidence supporting ceftazidime–avibactam activity against Acinetobacter baumannii remains very limited. Available data are largely restricted to in vitro studies evaluating potential synergistic effects in combination regimens [19-22]. CZA does not reliably inhibit the OXA-type carbapenemases (such as OXA-23, OXA-24/40, and OXA-58) that commonly mediate carbapenem resistance in *Acinetobacter baumannii*, and it is therefore not considered intrinsically active against this pathogen [23]. In addition, current EUCAST criteria do not provide validated susceptibility breakpoints for CZA against *Acinetobacter* species [21].

In our study, *Acinetobacter baumannii* represented only a very small proportion of infections (2 of 21 patients). In these critically ill children, CZA was used as a salvage strategy because no other active antimicrobial options were available, based on limited in vitro susceptibility data and clinical judgment. Therefore, observations related to *Acinetobacter baumannii* in this series should be interpreted cautiously and considered exploratory and hypothesis-generating rather than evidence of antimicrobial efficacy.

Accordingly, in our small subgroup of patients with PDR A. baumannii infections, CZA was used strictly as a salvage strategy in the absence of effective therapeutic alternatives. Clinical response was observed in one of two cases; however, given the limited mechanistic and clinical support, these findings should be interpreted cautiously and regarded as descriptive and hypothesis-generating rather than confirmatory of antimicrobial efficacy. Before the availability of CZA, alternative regimens such as double-carbapenem therapy combined with colistin or tigecycline were commonly used in pediatric intensive care, despite significant toxicity concerns and off-label use [24]. A previous publication from our center reported favorable outcomes with these strategies; however, safer and more targeted options were limited [25]. In this study, CZA was commonly administered as part of combination regimens, most frequently with meropenem or colistin, according to individualized clinical decision-making in critically ill patients. Nevertheless, due to the small sample size and heterogeneous clinical indications, this study was underpowered to assess the impact of combination therapy on outcomes, and no conclusions regarding the superiority of combination therapy over monotherapy can be drawn. In addition, treatment allocation (monotherapy versus combination therapy) reflected individualized clinical decision-making in critically ill children and likely introduced confounding by indication, as combination regimens were more frequently used in patients with more complex or severe infections. Therefore, regimen-based observations in this study should be interpreted as descriptive rather than comparative.

The duration of CZA therapy exceeded 14 days in 15 of 21 patients (71.4%). In pediatric clinical studies of CZA, treatment durations have generally ranged up to 14 days, depending on infection type and clinical response The expression [25] here should be removed Current pediatric practice does not mandate fixed treatment durations but emphasizes response-guided therapy, with prolonged courses reserved for persistent infection, delayed microbiological clearance, or clinical instability [26, 27].

In our study, extension beyond 14 days was primarily driven by persistent bacteremia, ongoing fever, hemodynamic instability, device-associated infections, or profound immunosuppression. In critically ill children with severe MDR/PDR infections, antimicrobial duration in our unit has historically been individualized based on clinical evolution rather than predefined thresholds. Importantly, extended CZA therapy in this series was not associated with increased drug-related adverse events or resistance emergence, consistent with available pediatric pharmacokinetic and safety data [11]. Similar variability in treatment duration has been reported in previous observational studies of ceftazidime–avibactam therapy, where median durations around 14–16 days and ranges extending beyond 30 days have been described in severe Gram-negative infections, reflecting the need for individualized treatment strategies in critically ill patients [28]. These findings suggest that, although prolonged therapy should not be routine, extension beyond 14 days may be justified in selected cases with ongoing clinical or microbiological instability.

Despite targeted therapy, four patients died from progressive multiorgan dysfunction. These non-survivors were younger and more frequently immunocompromised, suggesting that host-related factors played a major role in adverse outcomes rather than treatment failure. Resistance emergence during therapy was observed in a single case, a rare but recognized phenomenon attributed to mechanisms such as KPC Ω-loop mutations and porin alterations [29]. Although resistance remains uncommon among Enterobacterales, it is clinically significant and underscores the importance of antimicrobial stewardship, adequate source control, and close microbiological surveillance [30–32].

Nearly half of the patients required isolation due to colonization or infection with carbapenem-resistant organisms. Prolonged hospitalization and prior antimicrobial exposure were common, necessitating strict adherence to infection-control bundles. Although isolation status was not significantly associated with survival, robust infection-control and stewardship practices remain essential to preserve CZA efficacy in pediatric intensive care settings [33–36].

Our findings are consistent with recent pediatric cohorts from Türkiye reporting moderate survival rates in children with MDR and PDR *K. pneumoniae* infections [35]. Unlike studies limited to a single pathogen or age group, our study represents a broader PICU population, supporting the clinical relevance of CZA across diverse MDR and PDR infections.

This study has several limitations, including its retrospective design, small sample size, and the predominance of individualized treatment strategies, which limited robust comparative analyses. Additionally, genetic resistance mechanisms and therapeutic drug monitoring were not evaluated, although altered pharmacokinetics in critically ill children may influence treatment outcomes [11, 27]. Additionally, neonates were not included in this study, limiting the generalizability of our findings to neonatal intensive care populations, where pharmacokinetics, pathogen distribution, and treatment strategies may differ.

The use of CZA in critically ill children should be carefully integrated into antimicrobial stewardship frameworks. As carbapenem-resistant Enterobacterales are classified among the World Health Organization (WHO) priority pathogens, the increasing use of novel β-lactam/β-lactamase inhibitor combinations may create substantial selective pressure within hospital environments. Prolonged exposure to CZA, particularly in patients with extended ICU stays and high antimicrobial burden, may increase the risk of resistance emergence and facilitate the selection of resistant subpopulations. In addition, the ecological consequences of widespread or prolonged therapy should be considered, as selective pressure may influence local resistance epidemiology and contribute to the dissemination of resistant strains within intensive care units. In our PICU, the initiation of CZA was therefore restricted to carefully selected patients with limited therapeutic alternatives and was guided by multidisciplinary consultation involving pediatric infectious diseases specialists and microbiology teams, together with close microbiological monitoring during treatment.

In conclusion, CZA was used in critically ill children with MDR and PDR Gram-negative infections, with favorable clinical and microbiological outcomes observed in this study population. Mortality was primarily associated with host-related factors, including younger age and immunosuppression, rather than treatment failure, although resistance emergence was observed in a single case. Integration of CZA into pediatric antimicrobial stewardship frameworks, alongside strict stewardship and infection-control measures, may support treatment strategies in this high-risk population. Prospective multicenter studies are needed to further define optimal dosing, treatment duration, and stewardship strategies for CZA in critically ill children. The findings of this study may be particularly relevant in resource-limited settings, where access to newer antimicrobial agents and advanced therapeutic options may be restricted. In such contexts, real-world data from tertiary PICUs can provide valuable guidance for pragmatic and individualized treatment strategies in critically ill children.

## Data Availability

No datasets were generated or analysed during the current study.

## Abbreviations

CIM: Carbapenem inactivation method
CNS: Central nervous system
CRBSI: Catheter-related bloodstream infection
CZA: Ceftazidime–avibactam
EUCAST: European Committee on Antimicrobial Susceptibility Testing
ICU: Intensive care unit
KPC: *Klebsiella pneumoniae* Carbapenemase
MDR: Multidrug-resistant
MIC: Minimum inhibitory concentration
PDR: Pandrug-resistant
PELOD: Pediatric logistic organ dysfunction
PICU: Pediatric intensive care unit
PK: Pharmacokinetics
PRISM III: Pediatric risk of mortality score
VAP: Ventilator-associated pneumonia
